# Rapid Determination of Crude Protein Content in Alfalfa Based on Fourier Transform Infrared Spectroscopy

**DOI:** 10.3390/foods13142187

**Published:** 2024-07-11

**Authors:** Haijun Du, Yaru Zhang, Yanhua Ma, Wei Jiao, Ting Lei, He Su

**Affiliations:** 1 College of Mechanical and Electrical Engineering, Inner Mongolia Agricultural University, No. 36 Zhaowuda Road, Hohhot 010018, China; du15563886115@163.com (H.D.); leiting0401@163.com (T.L.); suhe0826@126.com (H.S.); 2 College of Horticulture and Plant Protection, Inner Mongolia Agricultural University, No. 36 Zhaowuda Road, Hohhot 010018, China; zhangyr0729@163.com; 3 The China Academy of Grassland Research, No. 120 Wulanchabu East Street, Saihan District, Hohhot 010018, China; jiaowei@caas.cn

**Keywords:** alfalfa, crude protein, FTIS, PLSR-CV

## Abstract

The crude protein (CP) content is an important determining factor for the quality of alfalfa, and its accurate and rapid evaluation is a challenge for the industry. A model was developed by combining Fourier transform infrared spectroscopy (FTIS) and chemometric analysis. Fourier spectra were collected in the range of 4000~400 cm^−1^. Adaptive iteratively reweighted penalized least squares (airPLS) and Savitzky–Golay (SG) were used for preprocessing the spectral data; competitive adaptive reweighted sampling (CARS) and the characteristic peaks of CP functional groups and moieties were used for feature selection; partial least squares regression (PLSR) and random forest regression (RFR) were used for quantitative prediction modelling. By comparing the combined prediction results of CP content, the predictive performance of airPLST-cars-PLSR-CV was the best, with an $R_{P}^{2}$ of 0.99 and an RMSEP of 0.053, which is suitable for establishing a small-sample prediction model. The research results show that the combination of the PLSR model can achieve an accurate prediction of the crude protein content of alfalfa forage, which can provide a reliable and effective new detection method for the crude protein content of alfalfa forage.

## 1. Introduction

Alfalfa is regarded as the “king of forages” due to its high protein content, and the constantly evolving livestock industry has led to an increasing demand for high-quality alfalfa grass for animal feed [1]. In particular, Inner Mongolia is a major livestock farming base in China, which requires large quantities of high-quality forage to address the seasonal and regional shortages of feed resources. This has presented ongoing challenges for the quality assessment of forage grasses [2,3]. The feeding value of alfalfa is determined by the ratio of crude protein (CP) content to fiber content [4]. As a high-quality forage, the abundant protein in alfalfa can play an important role in improving the nutritional status, product quality, and yield of cattle and sheep [5]. However, the variety of alfalfa, the planting region, the harvesting time, the drying method, etc., all affect the CP content in alfalfa [6,7,8], which results in the inconsistent quality of alfalfa circulating in the market. Feeding livestock with alfalfa products low in CP content will prolong their growth cycle. Therefore, accurately evaluating the CP content of alfalfa is one way to assess the quality of forage products. The currently common chemical method for determining the nutritional value of forage is complex in operation, time-consuming, with certain risks to the operators, and requires the consumption of large amounts of reagents, leading to environmental pollution [9]. Therefore, finding a rapid, accurate, and efficient method to detect the nutritional value of alfalfa is of great significance for promoting the rapid development of the alfalfa industry [10].

The Fourier transform infrared spectroscopy (FTIS) technology has been widely used in the quality detection of agricultural products [11]. This is a fast, efficient, and non-destructive detection method [12,13]. He et al. utilized the Fourier transform near-infrared spectroscopy (FT-NIRS) technology and the TQAnalyst v9 software to accurately determine the CP content in alfalfa hay [9]. Díaz et al. proposed the use of near-infrared spectroscopy (NIR) to non-destructively detect the protein, starch, and other contents in rice, thereby classifying the rice flavor [14]. Lu et al. utilized FT-NIRS and partial least squares regression (PLSR) combined with cross-validation to establish a quantitative model, which achieved excellent results in predicting the soluble solids and titratable acidity of pears [15]. Sahachairungrueng et al. used PLSR to fit the NIR information with chemical properties and digestibility, generating a set of trained NIR models that can be used for rapid biomass measurement [16].

The application of machine learning algorithms to the processing and prediction of infrared spectral data can improve data understanding and quantification, and achieve relatively high accuracy [17]. Mishra et al. used PLSR analysis and infrared spectroscopy to quantitatively detect the protein content in rice, with a detection error of 0.349% [18]. Baath et al.’s work using NIRS technology employed support vector machines (SVMs) and least-squares SVMs to predict the crude protein content of soybean and velvet bean feed samples. The reported R^2^ values ranged from 0.92 to 0.98 [19]. Wang et al. used competitive adaptive reweighted sampling (CARS) to screen spectral features and used PLSR to establish prediction models for the non-destructive detection of protein and fat content in Chinese torreya, with coefficients of determination (R^2^) of 0.977 and 0.984, respectively [20]. Magali used a random forest regression (RFR) model to predict and estimate the nitrogen content of feed corn, demonstrating good predictive performance [21]. Tian et al. used PLSR to establish quantitative analysis models for the nutritional components of fresh and dried oat samples, enabling the rapid detection of the nutritional quality of oat forage silage [22]. However, there are few reports on the use of Fourier transform infrared spectroscopy and machine learning to construct quantitative analysis models for the CP content of alfalfa.

The present study aims to identify alfalfa from different regions and varieties in Inner Mongolia within the spectral range of 400~4000 cm^−1^ using FTIS. It also combines chemometric methods to conduct a physicochemical analysis of the CP content in alfalfa, exploring the relationship between content variation and spectral information. This aims to provide technical guidance for the accurate, rapid, and non-destructive detection of the CP content in alfalfa.

## 2. Materials and Methods

### 2.1. Materials

The alfalfa grass came from the Inner Mongolia Agricultural University experimental base (located in Saihan District, Hohhot, Inner Mongolia, China) and the Hohhot alfalfa planting base (located in Hulin’ger County, Hohhot, Inner Mongolia, China). After artificial harvesting, the fresh alfalfa had a moisture content of approximately 70% to 80%; it was placed in a well-ventilated and shaded factory (temperature around 26 °C, relative humidity 12%), and turned twice a day. Using a portable moisture content meter (High-precision MS-H forage moisture meter, measurement range 0~84%, accuracy range 1%, Qingdao Tuoke Instrument Co., Ltd., Qingdao, China), the moisture content was determined, and when the moisture content of the alfalfa was less than 10%, the alfalfa grass from different areas, planting conditions, and varieties was crushed into granules to obtain the alfalfa grass samples. The prepared 90 grass samples were placed in a drying oven at 60 °C until they reached a constant weight.

### 2.2. Chemical Analysis Methods

The CP content in the alfalfa was determined using the Kjeldahl method according to the Chinese national standard GB 5009.5-2016 [23]. In total, 1 g of the thoroughly mixed solid sample, accurate to 0.001 g, was weighed and transferred to the digestion flask. Then, 0.4 g of copper sulfate, 6 g of potassium sulfate, and 20 mL of sulfuric acid were added and digested in the digestion furnace. The digestion temperature was 420 °C, and the duration was 1 h. After cooling, 50 mL of purified water was added, and then an automated Kjeldahl nitrogen analyzer (K9860 Fully Automatic Kjeldahl Nitrogen Analyzer—Hunan Honglin Scientific Instrument Co., Ltd., Changsha, China) was used to conduct the titration process and output the crude protein content result. The crude protein content range of alfalfa grass was 9.00% to 22.04%.

### 2.3. The Spectral Curve Measurement of the Sample

**Figure 1:**
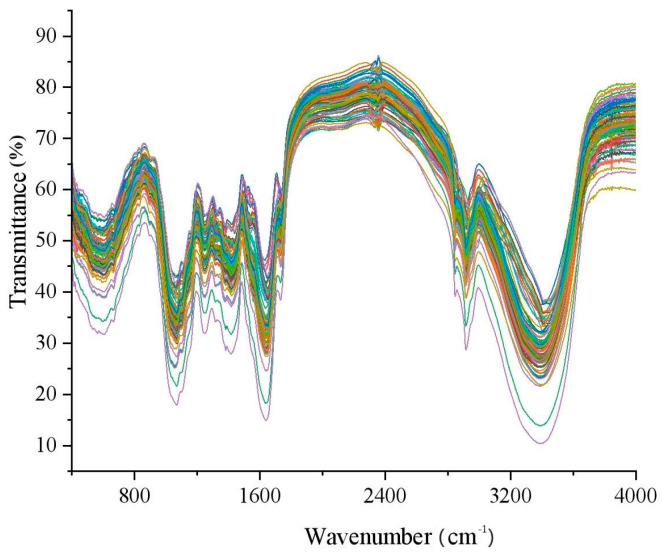
FTIS of alfalfa samples.

The analytical instrument used was the FTIR-1500 Fourier Transform Infrared Spectrometer (Zhengzhou oda Instrument Co., Ltd., Zhengzhou, China), which can collect spectra in the range of 4000~400 cm^−1^ with a resolution better than 1 cm^−1^ and 16 scans. Prior to sample scanning, the instrument was preheated for 0.5 h. Approximately 0.02 g of dry powder and 2.5 g of potassium bromide (KBr) dried at 80 °C for 10 h were ground for more than 10 min using a quartz mortar and pestle. Then, 0.18 g of prepared sample was pressed into a test sample using a press and placed in the spectrometer for scanning to obtain the spectrum. The accompanying Fourier transform infrared spectroscopy software was used to collect the raw spectral data. Before each sample spectrum collection, (air) background collection was performed. Each sample was randomly measured 10 times, and the spectral curve of the sample was constructed using the average method. The FTIS of the sample is shown in Figure 1. The spectral data have 10,329 characteristic points.

### 2.4. Spectrum Preprocessing

The FTIS data of alfalfa were preprocessed to eliminate noise and outliers in the spectral data [24]. The adaptive iteratively reweighted penalized least squares (airPLS) method was used to correct the entire spectrum [25]. AirPLS is an improved method based on weighted least squares to weigh the original model. AirPLS estimates the baseline of the original signal, controls the smoothness of the baseline, and eliminates the baseline drift in the spectral data, thereby improving the accuracy of baseline estimation [26,27]. In this study, the adaptive iteratively reweighted penalized least squares (airPLS) algorithm was used to preprocess the Fourier transform spectrum of alfalfa, with lambda set to 10 × 10^9^, the order of the difference of penalties set to 2, the weight exception proportion at both the start and end set to 0.05, the asymmetry parameter for the start and end set to 0.5, and the maximum iteration times set to 20. The Savitzky–Golay (SG) algorithm was used to smooth and differentiate the signal data to improve the spectral quality and enable accurate quantitative analysis [28,29]. After applying the smoothing algorithm, the min-max normalization method was further used to process the data, as these preprocessing steps are crucial for minimizing the impact of light scattering on the development and validation of classification models [30].

### 2.5. Spectral Feature Selection

Spectral data often require feature selection to overcome the challenge of a large number of variables in the data, and to remove wavelength features that are not related to the protein. Feature selection aims to identify the most relevant and informative features, which can improve model accuracy, reduce computational cost, and enhance interpretability [31]. Various feature selection techniques can be utilized, such as peak identification, cluster analysis, statistical methods, and a more advanced method, CARS [32]. The CARS algorithm can adaptively select the optimal subset from a large number of variables, significantly reducing model complexity while improving model prediction accuracy [33].

These techniques assess the correlation between each wavelength and the target variable, considering factors such as the signal-to-noise ratio, spectral characteristics, and relevance to potential physical or chemical processes. In the current research, CARS is used to select a subset of important features from the spectra, which can improve model accuracy by removing redundant or noisy information and prevent overfitting, especially in small data sets [34].

The PLS model [35] and the random forest model were constructed using a combination of preprocessing algorithms and different feature wavelength selection algorithms to estimate the crude protein content of alfalfa. The model prediction results were evaluated based on the coefficient of determination ($R_{c}^{2}$) and root mean square error (RMSEC) of the calibration set, as well as the coefficient of determination ($R_{p}^{2}$) and root mean square error (RMSEP) of the prediction set. An $R_{c}^{2}$ and $R_{p}^{2}$ closer to 1 indicate better model stability, goodness of fit, and accuracy, while RMSEC and RMSEP closer to 0 suggest smaller model errors and better predictive capability [36].

(1) $$R_{C}^{2}=1-\frac{\sum {\left(C_{i}-\hat{C_{i}}\right)}^{2}}{\sum {\left(C_{i}-C_{n}\right)}^{2}},$$

(2) $$R_{P}^{2}=1-\frac{\sum {\left(C_{i}-\hat{C_{i}}\right)}^{2}}{\sum {\left(C_{i}-C_{m}\right)}^{2}},$$

(3) $$RMSEC=\sqrt{\frac{\sum {\left(C_{i}-\hat{C_{i}}\right)}^{2}}{n}},$$

(4) $$RMSEP=\sqrt{\frac{\sum {\left(C_{i}-\hat{C_{i}}\right)}^{2}}{m}},$$

where $C_{i}$ is the *i*-th measured value of the reference method; $C_{i}$ is the *i*-th predicted value of the model; $C_{n}$ is the mean value of the measured values of n samples; $C_{m}$ is the mean of the predicted values of m samples; m is the number of samples in the prediction sets; and n is the number of samples in the calibration sets.

In this work, all data analyses, including spectral pretreatment, wavelength selection, and PLS modeling, were performed in the Python 3.11 software.

## 3. Results and Discussion

### 3.1. Spectral Feature Analysis

The Fourier transform infrared transmittance spectra of alfalfa samples in the range of 400–4000 cm^−1^ are shown in Figure 1. The figure depicts the average raw transmittance spectra of 90 alfalfa samples. The spectra exhibit a consistent pattern with essentially similar curve shapes. Relatively strong absorption peaks are observed around 593, 600, 665, 1053, 1102, 1246, 1381, 1417, 1630, 2915, and 3394 cm^−1^. The absorption peak around 3394 cm^−1^ is associated with the asymmetric stretching frequency of the NH2 group, while the absorption peak around 1630 cm^−1^ is related to the bending vibration frequency of the NH2 group. The absorption peak at 1246 cm^−1^ is associated with the stretching vibration of the C-N group. Additionally, the absorption peak around 1246 cm^−1^ is related to the stretching vibration of the COOH group, and the absorption peak at 1417 cm^−1^ is associated with the in-plane bending vibration of the COOH group. The peaks at 600 and 650 cm^−1^ are related to the vibration frequency of the C-OH group.

Based on the characteristic absorption peak spectral frequencies of functional groups and moieties (FG&M) present in crude protein, absorption peak value data at corresponding frequencies can be extracted as characteristic values for crude protein content. This method can serve as a means of feature extraction for analyzing and evaluating crude protein content.

### 3.2. FTIS Preprocessing

**Figure 2:**
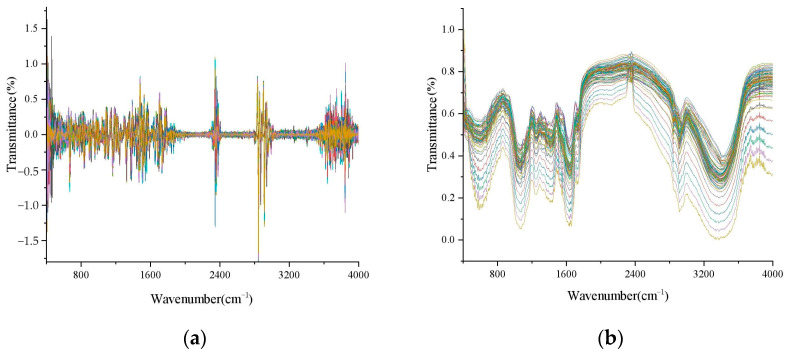
The preprocessed results: (**a**) AirPLS; (**b**) SG.

The application of the airPLS method to the original spectral data and the correction preprocessing of the entire spectrum resulted in the transmittance spectrum shown in Figure 2a. The application of the SG algorithm and normalization to the correction preprocessing of the entire spectrum resulted in the transmittance spectrum shown in Figure 2b.

The 90-sample spectra are divided into training, validation, and prediction sets according to a 7:1.5:1.5 ratio, resulting in 63 training samples, 13 validation samples, and 14 prediction samples. Based on Table 1, the use of the PLSR model and feature screening preprocessing methods resulted in a very small coefficient of determination in the final results, and the root mean square errors were quite large, indicating that the feature vector contained too many features, leading to significant interference and noise. Among the preprocessing methods, the SG algorithm for the entire spectrum performed the worst, and therefore this method was excluded from the preprocessing of the spectral data.

### 3.3. Effective Wavelength Selection

The selection of characteristic wavelengths in spectral data is based on the comparative results of pretreatment research and the selection of the most effective pretreatment procedure. However, even after preprocessing the actual spectrum, the prediction performance is still not good, due to the large amount of data. Therefore, it is necessary to perform dimensionality reduction on the full-band spectrum after airPLS.

**Figure 3:**
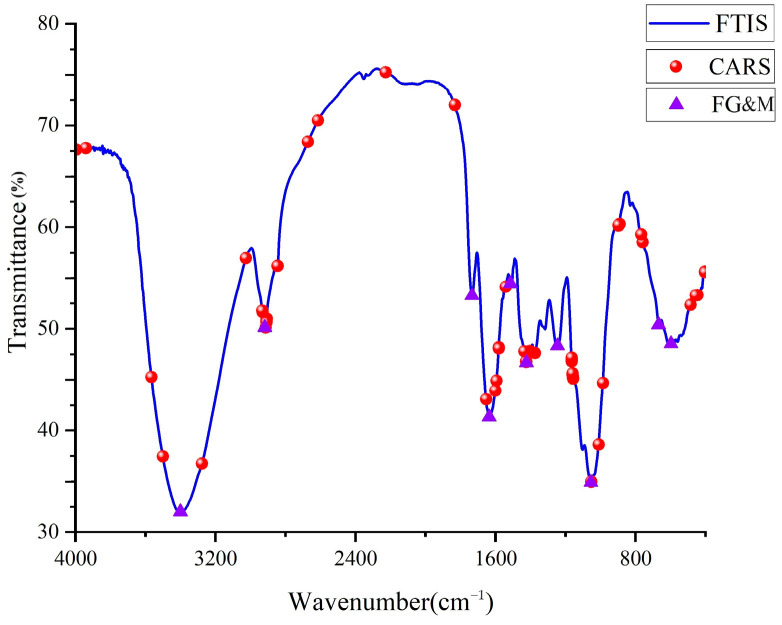
Using CARS and FG&M to select features related to the spectral characteristics of crude protein.

The wavelength results obtained using the CARS method are shown in Figure 3. Through two methods of screening feature points, it was found that there are overlapping feature points around the absorption peaks at 2915, 1633, 1512, 1420, and 1242 cm^−1^ wavenumber. The absorption band in the 3000~2800 cm^−1^ region is most likely attributed to C-H and NH3, which can be referred to as free amino acids and phenols. The region of 1700~1550 cm^−1^ is the characteristic peak for proteins [37]. The 1750–940 cm^−1^ wavenumber range corresponds to the C-OH group and C-O stretching vibrations in the phenol structure. The CARS feature selection method, when dealing with large-scale data features, converges through each iteration and gradually eliminates irrelevant or weakly correlated features. However, more is needed to guarantee the complete elimination of weakly correlated features. In fact, certain spectral positions exhibit significant noise that cannot be sufficiently reduced through data preprocessing methods, resulting in the retention of some weakly correlated feature points.

**Figure 4:**
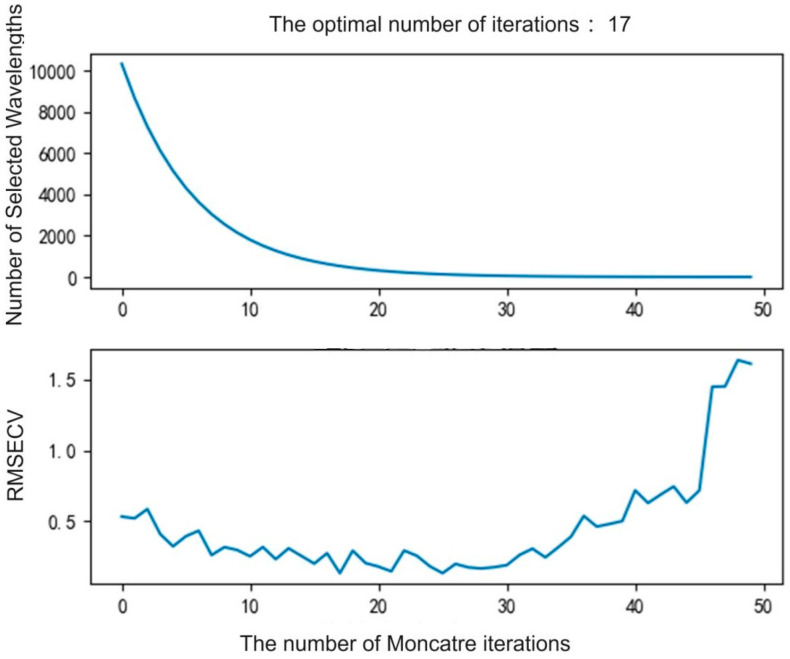
The results of the CARS method for extracting feature values.

The results of the CARS method for extracting feature values are shown in Figure 4. As the number of iterations increases, the number of selected wavelengths gradually decreases and becomes stable when the number of iterations approaches 20. The RMSECV value gradually decreases in the early stage, and then reaches the minimum value when the number of iterations is close to 17, after which the RMSECV value begins to rise. This indicates that at the 17th iteration, the CARS method has achieved the best balance between the number of selected wavelengths and the predictive performance of the model. This suggests that the CARS method gradually screens out the most representative wavelengths during the iteration process, and ultimately retains fewer but more informative wavelengths.

The PLSR models developed using feature wavelengths selected in various ways are shown in Table 2. The PLSR model developed using feature wavelengths selected by CARS exhibited superior predictive performance for protein, with a prediction set $R_{P}^{2}$ of 0.89 and an RMSEP of 0.75. The PLSR model developed using feature wavelengths selected based on protein absorption peaks showed a moderate predictive ability for protein, with a relatively low coefficient of determination. The RFR models developed using different feature selection methods all exhibited excellent predictive performance for CP. Among them, the RFR model developed using feature wavelengths selected by CARS showed the best predictive performance for CP, with a prediction set $R_{P}^{2}$ of 0.96 and an RMSEP of 0.41, indicating its high capability in predicting CP content.

The feature extraction using airPLS-CARS and the use of RFR for predicting the CP content of alfalfa demonstrated excellent predictive capabilities. The $R_{c}^{2}$ = 0.96 and $R_{p}^{2}\;$= 0.96 values were higher than those of the PLSR model, and the RMSE was comparable, with RMSE values being smaller than those of the PLSR model. This indicates that the RFR model has more stable predictive performance.

**Figure 5:**
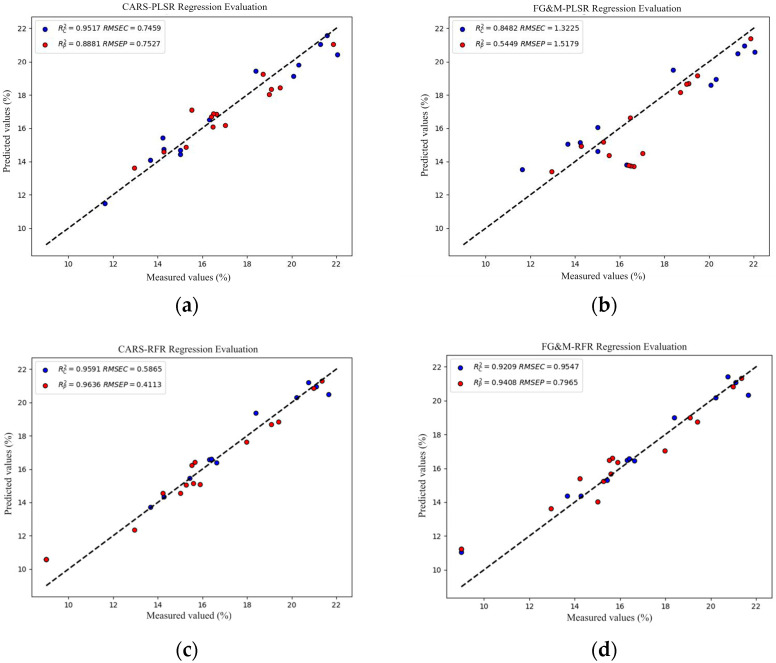
Predicted CP content: (**a**) airPLS-CARS-PLSR; (**b**) airPLS-FG&M-PLSR; (**c**) airPLS-CARS-RFR; and (**d**) airPLS-FG&M-RFR.

Alfalfa CP content was predicted using FG&M and RFR, with an $R_{P}^{2}$ of 0.94 and an RMSEP of 0.79, which is slightly lower in accuracy than the CARS feature extraction method, but the number of feature values is 4/11 of the CARS extracted feature values. It is inevitable that water molecules cannot be completely removed from the samples and the manufacturing environment, and potassium bromide itself will absorb water molecules from the air, so the measured spectra show an absorption peak around 3400 cm^−1^ due to water, which interferes with the peak feature values of NH2 in this region [38]. The CARS method avoids the 3400 cm^−1^ feature peak and instead selects feature peaks at 3368, 3445, and 3477 cm^−1^ around it, which can avoid interference from water. The prediction results of airPLS-CARS-PLSR, airPLS-FG&M-PLSR, airPLS-CARS-RFR, and airPLS-FG&M-RFR are shown in Figure 5.

### 3.4. Discussion of Final Models

**Figure 6:**
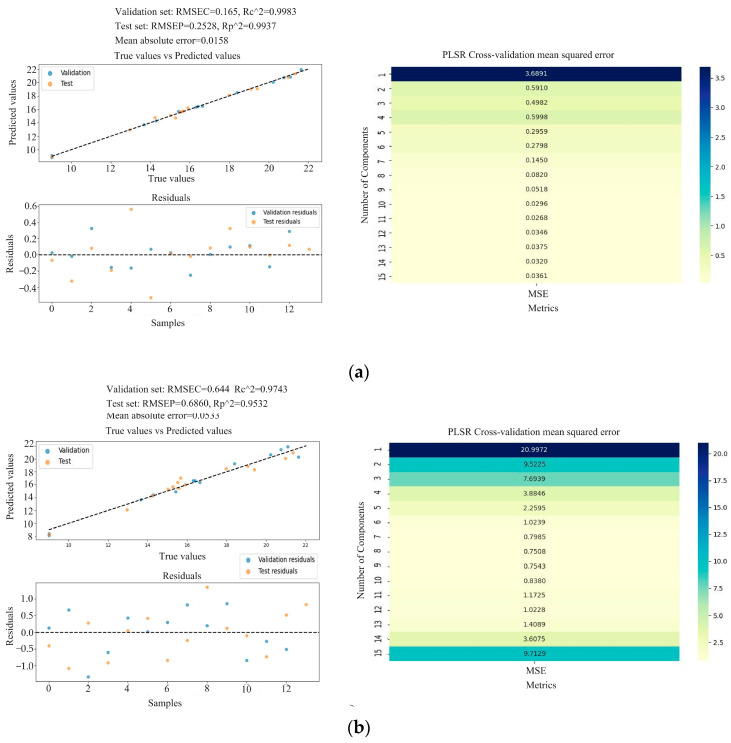
Optimization prediction results: (**a**) airPLS-CARS-PLSR-CV; (**b**) airPLS-FG&M-PLSR-CV.

**Figure 7:**
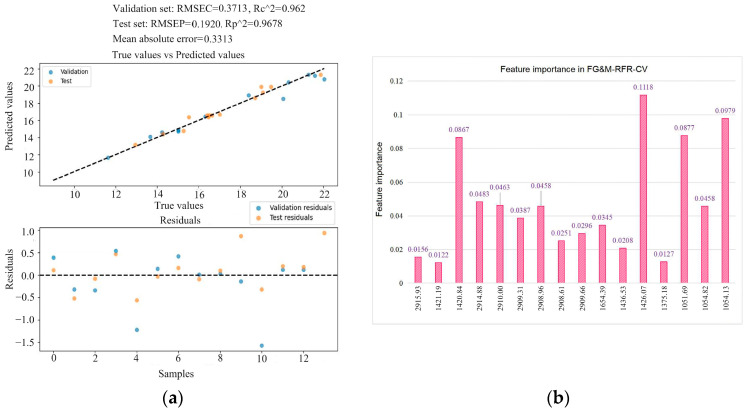
The optimized results of the RFR model based on FG&M-selected features: (**a**) predictive analysis results; (**b**) feature importance in FG&-MRFR-CV.

**Figure 8:**
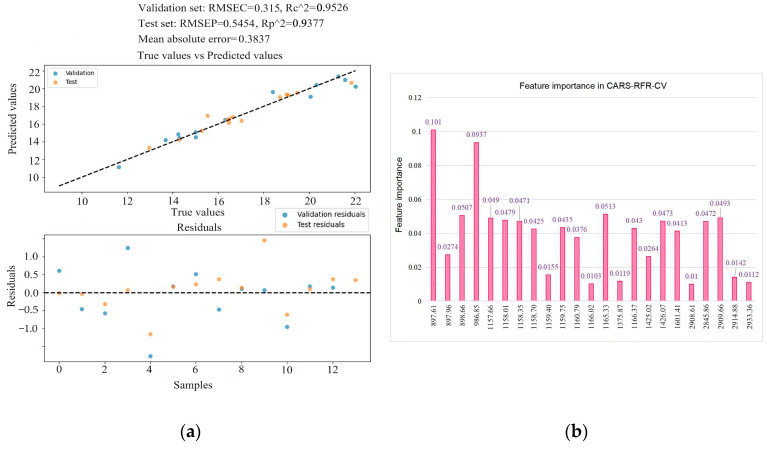
The optimized results of the RFR model based on CARS-selected features: (**a**) predictive analysis results; (**b**) feature importance in CARS-RFR-CV.

Through the feature vectors obtained using different feature screening methods, the protein is predicted by the PLSR and RFR models, both of which have good predictive performance. Among them, the RFR prediction performance is better and more stable than that of the PLSR model. In order to obtain a relatively stable and better predictive performance, the cross-validation (CV) of K-fold Cross-Validation is introduced to find the optimal number of principal components, thereby optimizing the predictive performance of the PLSR prediction model [39]; Figure 6, Figure 7 and Figure 8 show the optimized PLSR and RFR optimization prediction results.

Among them, the PLSR model developed using the CARS-selected features has a predictive performance of $R_{P}^{2}\;$= 0.99 and RMSEP = 0.25 (as shown in Figure 6a), and the average of the absolute error between the prediction and the target is 0.0158. The heatmap of the optimal number of principal components shows that when the number of principal components is 11, the total RMSE of the model is minimized. The PLSR model developed using FG&M-selected features has a predictive performance of $R_{P}^{2}\;$= 0.95 and RMSEP = 0.69 (as shown in Figure 6b), and the average of the absolute error between the prediction and the target is 0.0533. The heatmap of the optimal number of principal components shows that when the number of principal components is eight, the total RMSE of the model is minimized.

The introduced CV and parameter optimization of the RFR model, as well as the automated hyperparameter tuning, can efficiently find the optimal parameter combination and thereby improve the overall performance of the model. Figure 6 shows the optimized RFR prediction results and optimization results. The RFR model developed using the FG&M-selected features has a prediction performance of $R_{p}^{2}$ = 0.96 and RMSEP = 0.19 (as shown in Figure 7a), with an average absolute error of 0.33 between the predictions and the targets. The random forest feature importance analysis reveals the key feature peaks in the model, as shown in Figure 7b, with the critical peaks at 1426.07, 1054.13, 1051.69, and 1420.84 cm^−1^. The optimized model parameters are as follows: max_depth: 10; max_features: ‘sqrt’; min_samples_leaf: 1; min_samples_split: 2; n_estimators: 15.

The developed RFR model based on the features selected by CARS for protein prediction performance gives an R-squared value of 0.93 and an RMSEP of 0.54 (as shown in Figure 8a), with an average absolute error of 0.38 between the predicted and target values. Through the random forest feature importance analysis (as shown in Figure 8b), the key features in the model are identified as 897.61, 986.85, 898.66, 1157.66, 1165.53, and 2909.66 cm^−1^. The optimized model parameters are as follows: max_depth: 10; max_features: ‘sqrt’; min_samples_leaf: 1; min_samples_split: 2; n_estimators: 15. The overall performance of the CARS-RFR-CV model is lower than that of the FG&M-RFR-CV model. The RFR model is suitable for predicting a small number of features, as an excessive number of feature variables can hinder its computational process and interfere with the calculation results.

The final decision coefficient for the predictive performance of the crude protein content prediction model is as follows: airPLS-CARS-PLSR-CV > airPLS-FG&M-RFR-CV > airPLS-FG&M-PLSR-CV > airPLS-CARS-RFR-CV. The root mean square error of the prediction is as follows: airPLS-FG&M-RFR-CV < airPLS-CARS-PLSR-CV < airPLS-CARS-RFR-CV < airPLS-FG&M-PLSR-CV. The airPLS-CARS-PLSR-CV model has the best predictive performance and relatively stable predictive results for predicting crude protein content, with the prediction accuracy as the primary evaluation metric and a smaller root mean square error. CARS can select wavelength variables with high weights and delete wavelength variables with low weights to improve model performance [40]. The feature wavelength variables screened out based on the spectral characteristics of FG&M of proteins have certain predictive ability, but they may ignore some key hidden feature wavelengths, causing a decline in predictive performance. RFR is a method that randomly selects the feature set size, evaluates the size of each decision tree, and then estimates the best size based on the decision tree prediction. It is very fast because it only considers a few candidates. The RFR model does not require too much feature processing and has strong robustness to outliers and interference values [41,42].

## 4. Conclusions

This study established a promising airPLS-CARS-PLS-CV model and compared its predictive performance and model robustness to the PLSR-based prediction model. Among the four models, the airPLS-CARS-PLS-CV model reduced the RMSEP for crude protein by 88.89%, 88.87%, and 58.33% compared to the full PLSR model, the airPLS model, and the airPLS-CARS-PLS model, respectively. These results indicate that the wavelength selection procedure can effectively improve the predictive performance of the model. Feature selection using the FG&M feature wavelengths can reduce the number of features, but the predictive performance is slightly lower than that of the airPLS-CARS-PLS-CV model. In the case of a small number of feature values, the model based on airPLS-FG&M-RFR-CV has better predictive capability and better stability. Overall, the results confirm the feasibility of using Fourier transform infrared spectroscopy for the rapid determination of crude protein in alfalfa.

## Figures and Tables

**Table 1:** Comparison of performance of PLSR models with different pretreatment methods.

Pretreatment	Calibration Set		Prediction Set	
	RMSEC	$R_{c}^{2}$	RMSEP	$R_{P}^{2}$
without	2.23	0.35	2.55	0.37
airPLS	2.22	0.35	2.14	0.55
SG	2.23	0.35	2.55	0.36

**Table 2:** Comparison of performance of PLSR and RFR models with different feature selection methods.

Model	Pretreatment	Number	Calibration Set		Prediction Set	
			RMSEC	$R_{c}^{2}$	RMSEP	$R_{P}^{2}$
PLSR	airPLS-CARS	55	0.74	0.95	0.75	0.89
	airPLS-FG&M	20	1.32	0.85	1.51	0.54
RFR	airPLS-CARS	55	0.58	0.96	0.41	0.96
	airPLS-FG&M	20	0.95	0.92	0.79	0.94

## Data Availability

The original contributions presented in the study are included in the article, further inquiries can be directed to the corresponding author.
